# miR-141-3p is Poorly Expressed in Polycystic Ovary Syndrome and Correlates with Glucose and Lipid Metabolism

**DOI:** 10.1155/2021/2022938

**Published:** 2021-10-08

**Authors:** Lingye Fan, Chunyan Wang, Ping Zhan, Yaofang Liu

**Affiliations:** ^1^Department of Gynaecology, The Affiliated Hospital of Southwest Medical University, Luzhou 646000, Sichuan, China; ^2^Sichuan Treatment Center for Gynaecologic and Breast Diseases (Gynaecology), Luzhou 64600, Sichuan, China; ^3^Department of Reproductive Technology, The Affiliated Hospital of Southwest Medical University, 25 Taiping Street, Luzhou 646000, Sichuan, China

## Abstract

Polycystic ovary syndrome (PCOS) is a common endocrinopathy with high prevalence. miR-141-3p downregulation was reported in PCOS rats. This study intended to investigate miR-141-3p expression in serum of PCOS patients and its correlation with glucose and lipid metabolism. A total of 100 PCOS patients and 100 healthy controls were enrolled in this study. Clinical parameters and glucose and lipid indexes were analyzed. A 3-month fat reduction intervention was conducted to PCOS-obese patients. Expressions of miR-141-3p and PTEN were detected. WHR and levels of TG, HDL-C, FBG, FINS, HOMA-*β,* and HOMA-IR showed significant differences in PCOS patients. miR-141-3p was downregulated in PCOS patients. Area under ROC curve of miR-141-3p diagnosing PCOS-obese patients was 0.985 with specificity 95.35% and flexibility 93.33%. Levels of glucose and lipid metabolism indexes were increased while HDL-C level was decreased in miR-141-3p low expression group. Indexes of PCOS-obese patients were improved and miR-141-3p was upregulated after fat reduction intervention. PTEN was upregulated in PCOS patients and negatively correlated with miR-141-3p. In conclusion, miR-141-3p was downregulated in PCOS patients and had higher diagnostic value on PCOS and associated with glucose and lipid metabolism. miR-141-3p might play a role in glucose and lipid metabolism in PCOS-obese patients by targeting PTEN.

## 1. Introduction

Polycystic ovary syndrome (PCOS) is a common endocrine disorder occurring to 5–10% reproductive-aged women [1, 2]. The clinical manifestations of PCOS are heterogeneous, which include hyperandrogenemia, anovulation, and infertility [3]. Major symptoms of PCOS consist of irregular menstruation, amenorrhea, hirsutism, acne, and polycystic ovary [4]. In addition to gynecological syndromes, PCOS also leads to metabolism disorder [5]. PCOS contributes to alterations of reproductivity, metabolism, and cosmetology and impairs the quality of life [6]. PCOS also increases the risks of diabetes, cerebrovascular and cardiovascular diseases, and endometrial cancer [7, 8]. The actual cause of PCOS remains unknown but factors such as genetic factors, exposure to high levels of androgen, epigenetic factors, and environmental factors play critical roles in the development of PCOS [9]. The proper diagnosis is critical for the long-term clinical management of PCOS.

microRNAs (miRNAs) are endogenous, short noncoding RNA molecules consisting of 21–25 nucleotides which regulate gene expression at the posttranscriptional level [10]. Recent studies have reported that miRNAs are associated with the occurrence of PCOS and may be involved in PCOS-mediated insulin resistance and hormone metabolism disorder [11, 12]. miRNAs are differentially expressed in PCOS patients and healthy women, which is related to the metabolic variables and sex hormone [13, 14]. It has been reported that miR-141-3p was downregulated in the ovary of mice with PCOS and miR-141-3p overexpression suppressed the apoptosis of granulosa cells by targeting death-associated protein kinase 1 (DAPK1) [15, 16]. Dang et al. elucidated the involvement of miR-141-3p in glycolipid metabolism and miR-141-3p knockdown repressed insulin response and glucose uptake of AML12 hepatocyte [17]. Recent study also elicited that peroxisome ATP binding cassette, subfamily D, member 2 (ABCD2), in osteoarthritis chondrocytes can affect miR-141 expression, downregulate the expression of acyl-CoA synthetase 4 (ACSL4), and thus induce the accumulation of very long chain fatty acids (VLCFAs) [18]. However, there is no report about miR-141-3p expression in serum of PCOS patients and its correlation with glucose and lipid metabolism.

Subsequently, 24 downstream target genes of miR-141-3p were screened using bioinformatics analysis. Among the 24 target genes, phosphatase and tension homolog (PTEN), as a negative regulator of insulin signaling and peripheral insulin sensitivity, was reported to be involved in glucose and lipid metabolism [19, 20]. Moreover, PTEN was upregulated in PCOS and the downregulation of PTEN suppressed the apoptosis of granulosa cells in PCOS [21, 22]. Yet the relation between miR-141-3p and PTEN in PCOS has not been reported so far. This study aimed to study the expression of miR-141-3p in serum of PCOS patients and its correlation with glucose and lipid metabolism and to provide reference for the evaluation of metabolism disorder in PCOS patients to achieve effective management and early prevention of PCOS-associated metabolic diseases and cardiovascular-related complications.

## 2. Material and Methods

### 2.1. Ethics Statement

This study was approved by the Ethics Committee of The Affiliated Hospital of Southwest Medical University. Participants were informed and consent forms were signed voluntarily.

### 2.2. Research Subjects and Sample Collection

First, a total of 100 cases of PCOS patients treated in the department of gynaecology of The Affiliated Hospital of Southwest Medical University during January 2019 to December 2020 were enrolled in this study. The PCOS patients were further assigned into PCOS-nonobese group (*N* = 57) and PCOS-obese group (*N* = 43) based on the body mass index (BMI) in the Asia-Pacific region from the International Obesity Task Force of World Health Organization (WHO) in 2000 which defines BMI ≥ 25 as obese [23]. The included patients were 20–35 years old and in accordance with the diagnostic criteria of PCOS revised by Rotterdam consensus in 2003 [24]. The exclusive criteria for PCOS patients were as follows: 1) diagnosis with hypothalamic or pituitary amenorrhea, thyroid dysfunction, premature ovarian failure, and other diseases causing ovulation disorder; 2) diagnosis with Cushing's syndrome, congenital adrenal hyperplasia, and other diseases leading to elevated androgen levels; 3) treatment with hormone therapy within 3 months before the enrollment; and 4) presence of malignant tumor and liver and kidney dysfunction. Next, 100 healthy women at childbearing age who took physical examination in The Affiliated Hospital of Southwest Medical University were enrolled as controls and assigned into control-nonobese group (*N* = 70) and control-obese group (*N* = 30). Healthy controls were 20–35 years old and with normal menstruation. The exclusive criteria for healthy controls were as follows: 1) clinical manifestation of hyperandrogenemia; 2) treatment with hormone therapy within 3 months before the enrollment; 3) a history of thyroid, pituitary, adrenal, and other diseases affecting endocrine; and 4) a history of severe medical diseases. Then, 2 mL peripheral venous blood sample was collected immediately after enrollment and centrifuged at 4°C at 2000 g for 10 min. The supernatant was transferred into an Eppendorf (EP) tube and stored at −80°C. The sample received no freeze-thaw cycle before experiment.

In addition, patients in the PCOS-obese group received 3-month intervention to achieve effective and sustainable weight control through low calorie (42.48 kJ/kg) and high protein content (35% protein, 45% carbohydrate) diet with limited energy intake. Aerobic exercise, for instance, brisk walking, jogging, and swimming combined with resistant exercise (such as sit-ups and squats for 30–40 min), was conducted for 3-4 times each week. The participants were followed up every week by telephone call.

### 2.3. Detection of Clinicopathological Characteristics

Clinical data (BMI, waist-to-hip ratio (WHR)) were measured and calculated by trained doctors. The levels of lipid metabolism markers (triglyceride (TG), total cholesterol (TC), low density lipoprotein cholesterin (LDL-C), high density lipoprotein cholesterin (HDL-C)) and glucose metabolism markers (fasting blood-glucose (FBG), fasting insulin (FINS)) were analyzed using Roche COBAS E601 automatic electrochemiluminescence immunoassay analyzer. Tosoh HLC-723 G8 glycosylated hemoglobin (HbAlc) analyzer was adopted to analyze HbAlc. The islet function markers homeostasis model assessment of beta cell function index (HOMA-*β* = 20 × FINS/(FBG-3.5)) and homeostasis model assessment of insulin resistance (HOMA-IR = FBG × FINS/22.5)) were calculated on the basis of HOMA model.

### 2.4. Reverse Transcription-Quantitative Polymerase Chain Reaction (RT-qPCR)

Total RNA was extracted using TRIzol reagent (Invitrogen, Carlsbad, CA, USA) and reverse transcribed into cDNA using PrimeScript RT reagent kit (Takara, Dalian, China). qPCR was conducted on ABI7900HT fast PCR real-time system (Applied Biosystems, Foster city, CA, USA) using SYBR® Premix Ex Taq^TM^ II (Takara). The reaction conditions were as follows: predenaturation at 95°C for 10 min and 40 cycles of denaturation at 95°C for 10 s, annealing at 60°C for 20 s, and extension at 72°C for 34 s with U6 as an internal control. The 2^−ΔΔCt^ method was employed for data analysis [25]. The primers were synthetized by Sangon Biotech (Shanghai) Co., Ltd. (Shanghai, China). Primer sequences are illustrated in Table 1.

### 2.5. Bioinformatics Analysis

The downstream target genes of miRNA were predicted through ENCORI (http://starbase.sysu.edu.cn/), RNAInter (http://www.rna-society.org/rnainter/), TargetScan (http://www.targetscan.org/vert_71/), and miRDB (http://www.mirdb.org/) websites and intersections were taken.

### 2.6. Dual-Luciferase Reporter Assay

The binding sites of miR-141-3p and phosphatase and tension homolog (PTEN) were predicted through ENCORI StarBase (http://starbase.sysu.edu.cn/index.php). The complementary sequence and mutant sequence of miR-141-3p and PTEN were amplified and cloned onto pmiR-GLO luciferase vectors (Promega, Madison, WI, USA) to construct wild-type plasmid (PTEN-WT) and corresponding mutant plasmid (PETN-MUT). The plasmids were cotransfected with mimic NC or miR-141-3p mimic (GenePharma, Shanghai, China) into HEK293T cells (Shanghai Institute of Biochemistry and Cell Biology, Chinese Academy of Sciences, Shanghai, China). Luciferase activity was detected after 48 h of transfection.

### 2.7. Enzyme-Linked Immunosorbent Assay (ELISA)

The PTEN content in serum was detected by ELISA according to a previous study [26]. The samples and standard samples were incubated with horseradish peroxidase- (HRP-) labeled antibodies. The plate was then thoroughly cleaned. Substrate TMB was used to develop color. Under the catalytic action of peroxidase, TMB became blue. Absorbance at 150 nm was detected using Multiskan™ FC microplate reader (Thermo Fisher Scientific, Waltham, MA, USA). The expression of each detection index was calculated using the standard curve.

### 2.8. Statistical Analysis

The SPSS 21.0 (IBM Corp., Armonk, NY, USA), GraphPad Prism 8 (GraphPad Software, San Diego, CA, USA), and MedCalc® version 15.0 (MedCalc Software Ltd., Ostend, Belgium) were used for data analysis and graphing. The sample size was estimated in advance using G. Power. Normal distribution was validated by Shapiro–Wilk (W test) test. Variable data were presented as mean ± standard deviation or the median. The unpaired *t*-test was used to compare two groups and differences among groups were compared with one-way analysis of variance (ANOVA), followed by Tukey's multiple comparisons test. The diagnostic value of miR-141-3p on PCOS was analyzed using receiver operating characteristic (ROC) curve. *P* < 0.05 was indicative of statistically significant difference.

## 3. Results

### 3.1. Comparative Analysis of the Clinical Data between PCOS Patients and Healthy Controls

In this study, 100 PCOS patients and 100 healthy controls were enrolled as research subjects. PCOS patients were further assigned into PCOS-nonobese group (*N* = 57) and PCOS-obese group (*N* = 43) while healthy controls were assigned into control-nonobese group (*N* = 70) and control-obese group (*N* = 30) according to the BMI in the Asia-Pacific region from the International Obesity Task Force of WHO in 2000 which defines BMI ≥ 25 as obese. The clinical baseline characteristics and glucose and lipid metabolism indexes between PCOS patients and healthy controls were comparatively analyzed. The result showed that WHR and levels of TG, HDL-C, FBG, FINS, HOMA-*β*, and HOMA-IR in PCOS-nonobese group were significantly different from the control-nonobese group (all *P* < 0.05), while age, BMI, and levels of TC, LDL-C, and HbAlc showed no statistical significance. Similarly, WHR and levels of TG, HDL-C, FBG, FINS, HOMA-*β,* and HOMA-IR in PCOS-obese group showed significant differences relative to the control-obese group (all *P* < 0.05), while age, BMI, and levels of TC, LDL-C, and HbAlc showed no statistical significance. Moreover, the result showed that compared with PCOS-nonobese group, BMI, WHR, and levels of TG, HDL-C, FBG, FINS, HOMA-*β,* and HOMA-IR in PCOS-obese group showed significant differences (*P* < 0.05, Table 2).

### 3.2. miR-141-3p Was Poorly Expressed in Serum of PCOS Patients and Possessed Higher Diagnostic Efficiency

**Figure 1:**
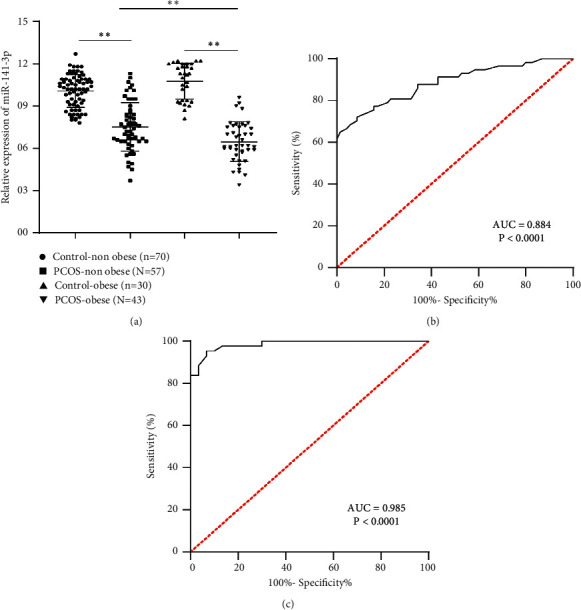
miR-141-3p was poorly expressed in serum of PCOS patients and possessed higher diagnostic efficiency. (a) miR-141-3p expression in serum of PCOS patients and healthy controls was detected by RT-qPCR; (b) diagnostic efficiency of miR-141-3p on PCOS-nonobese patients was analyzed using ROC curve; (c) diagnostic efficiency of miR-141-3p on PCOS-obese patients was analyzed using ROC curve. Data are expressed as mean ± standard deviation. Data in (a) were analyzed using one-way ANOVA, followed by Tukey's multiple comparisons test. ^*∗∗*^*P* < 0.01.

RT-qPCR showed that miR-141-3p expression was decreased in serum of the PCOS-nonobese group relative to the control-nonobese group; miR-141-3p expression was decreased in serum of the PCOS-obese group relative to the control-obese group; and miR-141-3p expression of PCOS-obese group was lower than that of PCOS-nonobese group (all *P* < 0.01, Figure 1(a)).

To further investigate the clinical diagnostic value of serum miR-141-3p expression on PCOS, the diagnostic efficiency of miR-141-3p was analyzed using ROC curve. The result showed that the area under the curve of PCOS-nonobese patients was 0.884 with specificity 98.57% and flexibility 64.91% (Figure 1(b)). The area under the curve of PCOS-obese patients was 0.985 with specificity 95.35% and flexibility 93.33% (Figure 1(c)). These results indicated that miR-141-3p possessed higher diagnostic efficiency on PCOS patients and the diagnostic efficiency on PCOS-obese patients surpassed that on PCOS-nonobese patients.

### 3.3. Correlation Analysis between miR-141-3p and Clinical Parameters

To further study the correlation between miR-141-3p and PCOS, the correlation between miR-141-3p expression and the clinical parameters of patients in PCOS-nonobese group and PCOS-obese group was analyzed using Pearson's coefficient. The result showed that the age, BMI, and LDL-C of PCOS-nonobese patients and PCOS-obese patients had no significant correlation with miR-141-3p expression, while the levels of glucose and lipid metabolism markers including TG, TC, FINS, HbAlc, HOMA-*β,* and HOMA-IR were negatively correlated with miR-141-3p expression (all *P* < 0.05, Table 3).

### 3.4. Changes of Clinical Parameters and miR-141-3p Expression in PCOS-Obese Patients before and after Intervention of Fat Reduction

**Figure 2:**
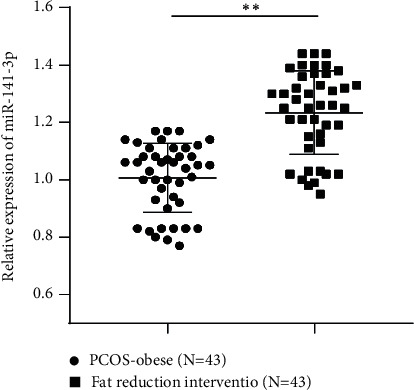
Changes of clinical parameters and miR-141-3p expression in PCOS-obese patients before and after intervention of fat reduction. miR-141-3p expression in serum of PCOS-obese patients before and after fat reduction intervention was detected by RT-qPCR. Data are expressed as mean ± standard deviation. Data were analyzed using *t*-test. ^*∗∗*^*P* < 0.01.

A total of 43 cases of PCOS-obese patients were received intervention of fat reduction for 3 months. Clinical parameters before and after intervention of fat reduction were compared and analyzed. The result showed that all the other metabolism parameters, except TG, TC, HDL-C, FBG, and HbAlc, were significantly decreased during the 3-month intervention (all *P* < 0.05, Table 4) and miR-141-3p expression was upregulated after intervention (all *P* < 0.01, Figure 2). The above results demonstrated that miR-141-3p might participate in the process of glucose and lipid metabolism in PCOS-obese patients.

### 3.5. PTEN Was Highly Expressed in Serum of PCOS Patients and Negatively Correlated with miR-141-3p

**Figure 3:**
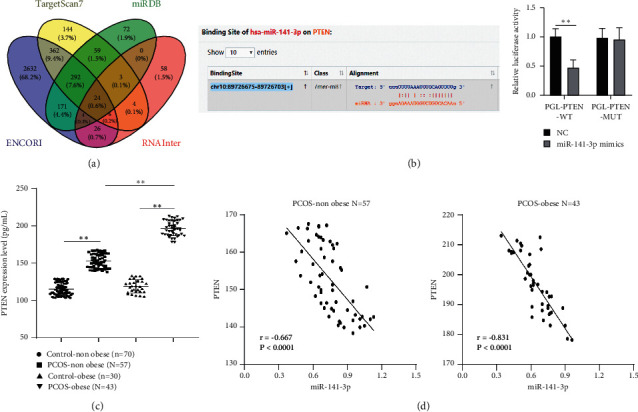
PTEN was highly expressed in serum of PCOS patients and negatively correlated with miR-141-3p. (a) Downstream target genes of miR-141-3p were predicted through TargetScan, StarBase, RNAInter, and miRDB database and intersections were taken; (b) target relation between miR-141-3p and PTEN was verified by dual-luciferase reporter assay; (c) PTEN expression in serum of PCOS patients was detected by ELISA; (d) correlation between miR-141-3p expression and PTEN expression in PCOS-nonobese patients and PCOS-obese patients was analyzed using Pearson's correlation analysis. Data are expressed as mean ± standard deviation. Data in (b) were analyzed using *t*-test and data in (c) were analyzed using one-way ANOVA, followed by Tukey's multiple comparisons test. ^*∗∗*^*P* < 0.01.

To explore the potential mechanism of miR-141-3p in the process of glucose and lipid metabolism in PCOS patients, the downstream target genes of miR-141-3p were predicted through TargetScan, StarBase, RNAInter (score ≥ 0.7), and miRDB database (score ≥70) and intersections were taken to obtain 24 target genes (Figure 3(a)). Among the 24 target genes, PTEN, as a negative regulator of insulin signaling and peripheral insulin sensitivity, was reported to be involved in glucose and lipid metabolism [19, 20]. Moreover, PTEN was upregulated in PCOS and the downregulation of PTEN suppressed the apoptosis of granulosa cells in PCOS [21, 22]. The target relation between miR-141-3p and PTEN was verified by dual-luciferase reporter assay (Figure 3(b)). Furthermore, ELISA showed that PTEN was upregulated in serum of PCOS-nonobese patients relative to control-nonobese patients; PTEN was upregulated in serum of PCOS-obese patients relative to control-obese patients; PTEN expression in serum of PCOS-obese patients was higher than that of PCOS-nonobese patients (all *P* < 0.01, Figure 3(c)). Pearson's correlation analysis showed that miR-141-3p expression was negatively correlated with PTEN expression in PCOS-nonobese patients and PCOS-obese patients (all *P* < 0.001, Figure 3(d)). These results elucidated that miR-141-3p might play a role in glucose and lipid metabolism in PCOS-obese patients by targeting PTEN.

## 4. Discussion

PCOS remains a major threat to the health of women around the world and carries considerable morbidity [27]. Interestingly, miRNAs may act as a biomarker to distinguish women with PCOS from healthy women according to previous study [28]. In the present study, we found that miR-141-3p was poorly expressed in serum of PCOS patients and had higher diagnostic value on PCOS and were associated with glucose and lipid metabolism disorder in PCOS. miR-141-3p might play a role in glucose and lipid metabolism in PCOS-obese patients by targeting PTEN. The results provided reference value for the clinical diagnosis of PCOS and the prediction of metabolic diseases and cardiovascular-related complications in PCOS patients.

In this study, the results showed that WHR and levels of TG, HDL-C, FBG, FINS, HOMA-*β,* and HOMA-IR in PCOS-nonobese patients and PCOS-obese patients presented significant differences relative to the healthy nonobese participants and healthy-obese participants. The data were also significantly different between PCOS-obese patients and PCOS-nonobese patients. miR-141-3p has been identified as being associated with PCOS [29]. In this study, the result showed that miR-141-3p expression was decreased in serum of PCOS patients. miR-141-3p expression in serum of PCOS-obese patients was lower than that of PCOS-nonobese patients. The result was consistent with a previous study elucidating that miR-141-3p was notably decreased in the ovaries of PCOS rats [16]. Furthermore, miR-141-3p had higher diagnostic value on PCOS-obese patients. The evaluation of miRNA and protein expression profiles in visceral adipose tissues from obese and nonobese females by Valentina et al. showed that miR-141 may increase glucose uptake and triglyceride synthesis by upregulating protein level of tyrosine 3-monooxygenase/tryptophan 5-monooxygenase activation protein gamma [30]. Collectively, miR-141-3p was poorly expressed in serum of PCOS patients and possessed higher diagnostic value on PCOS.

miRNAs are correlated with glucose, insulin, HOMA, and TG and HDL levels [31]. In this study, the levels of glucose and lipid metabolism markers including TG, TC, LDL-C, FBG, FINS, HbAlc, HOMA-*β*, and HOMA-IR were increased while HDL-C level was decreased in patients with low expression of miR-141-3p. miR-141-3p was reported to play a role in glucose transport and lipid metabolism [30]. Taken together, miR-141-3p was associated with glucose and lipid metabolism indexes in PCOS patients. Lifestyle adjustment such as diet control and exercise is the primary treatment of PCOS [32]. Hence, a 3-month fat reduction intervention was carried out on PCOS-obese patients. The result showed that all the other metabolism parameters, except TG, TC, HDL-C, FBG, and HbAlc, were significantly decreased during the 3-month intervention and miR-141-3p expression was significantly increased after intervention. Kim et al. have reported that miR-141-3p suppressed insulin sensitivity and glucose uptake of hepatocytes and expression of exosomal miR-141-3p was decreased in obesity-induced mice [33]. According to the study of Park et al., peroxisome ABCD2 could affect the expression of miR-141 in chondrocytes in osteoarthritis and reduce the expression of the direct regulator of lipid metabolism ACSL4 and thus induce the accumulation of very long chain fatty acids (VLCFAs) [18]. The tyrosine 3-monooxygenase/tryptophan 5-monooxygenase activation protein (YWHAz) gamma pathway is associated with glucose and lipid metabolism and could pose influence on glycolysis, gluconeogenesis, and fatty synthesis. Capobianco et al. evaluated the visceral adipose tissues of obese and nonobese females and the bioinformatics information of miRNA and protein expression profiles revealed that miR-141 increases glucose uptake and synthesis of triacylglycerol possibly by upregulating YWHAG [30]. In summary, miR-141-3p might participate in glucose and lipid metabolism in PCOS-obese patients.

To further explore the downstream mechanism of miR-141-3p in glucose and lipid metabolism, PTEN, target gene of miR-141-3p, was obtained through prediction and intersection. PTEN was reported to be increased in follicular fluid of patients with PCOS and ovarian cancer cell line [21]. Also, PTEN participated in the regulation of hepatic glucose and lipid metabolism [19]. In this study, the target relation between miR-141-3p and PTEN was verified by dual-luciferase reporter assay. The study result also showed that PTEN was upregulated in serum of PCOS-nonobese patients relative to healthy-nonobese participants; PTEN was upregulated in serum of PCOS-obese patients relative to healthy-obese participants; PTEN expression in serum of PCOS-obese patients was higher than that of PCOS-nonobese patients. miR-141-3p remarkably reduced the PTEN level and PTEN expression was inversely correlated with miR-141-3p expression according to previous studies [34, 35]. Our study further confirmed that miR-141-3p was negatively correlated with PTEN. Collectively, miR-141-3p might play a role in glucose and lipid metabolism in PCOS-obese patients by targeting PTEN.

In summary, miR-141-3p was poorly expressed in serum of PCOS patients and associated with glucose and lipid metabolism in PCOS, indicating a higher diagnostic value on PCOS. However, this study has several limitations. Firstly, a relatively small group was enrolled in this study. Secondly, the indexes of endocrine (including follicle-stimulating hormone (FSH), luteinizing hormone (LH), total/free testosterone, sex hormone-binding globulin (SHBG), and dehydroepiandrosterone (sodium) sulfate (DHEA-SO4)) were not included in the analysis. Meanwhile, it would be significant to analyze the correlation between the endocrine indexes and miR-141-3p expression. Finally, this study simply elucidated the target relation between miR-141-3p and PTEN while deeper exploration is required for the specific mechanism of miR-141-3p in glucose and lipid metabolism in PCOS. The focus of future studies lies on rests with the molecular mechanism of miR-141-3p and PTEN in the glucose and lipid metabolism in PCOS-obese patients, other possible target genes of miR-141-3p, and their regulatory mechanisms in the glucose and lipid metabolism in PCOS-obese patients.

## Figures and Tables

**Table 1:** Primer sequences.

Gene	Forward 5′–3′	Reverse 5′–3′
miR-141-3p	GCGGAAAGAGGCCCCG	AGTGCAGGGTCCGAGGTATT
U6	ATTGGAACGATACAGAGAAGATT	GGAACGCTTCACGAATTTC

*Note*. miR, microRNA.

**Table 2:** Comparative analysis of the clinical data between PCOS patients and healthy controls.

Parameters	Control-nonobese (*N* = 70)	PCOS-nonobese	Control-obese	PCOS-obese	*P*_*a*_	*P*_*b*_	*P*_*c*_
		(*N* = 57)	(*N* = 30)	(*N* = 43)			
Age (years)	27.25 (25.07–30.38)	26.33 (24.31–30.19)	27.01 (24.22–30.14)	27.42 (24.53–30.60)	0.063	0.642	0.289
BMI (kg/m^2^)	21.00 (19.12–23.68)	20.75 (18.67–24.71)	27.76 (24.84–31.03)	28.55 (25.43–30.60)	0.693	0.253	<0.001
WHR	0.70 (0.62–0.81)	0.74 (0.65–0.82)	0.85 (0.71–1.00)	0.92 (0.82–1.02)	0.047	0.001	<0.001
TG (mmol/L)	1.18 (0.70–1.88)	1.33 (0.92–2.13)	1.36 (0.78–2.00)	1.51 (1.04–2.21)	0.001	0.003	0.014
TC (mmol/L)	1.10 (0.66–1.73)	1.13 (0.80–1.76)	1.19 (0.59–1.84)	1.30 (0.91–1.89)	0.232	0.319	0.334
LDL-C (mmol/L)	2.26 (1.85–2.85)	2.41 (1.89–2.85)	2.39 (1.82–3.01)	2.52 (1.99–2.91)	0.269	0.306	0.062
HDL-C (mmol/L)	1.09 (0.62–1.75)	0.75 (0.37–1.46)	1.24 (0.66–1.88)	0.64 (0.17–1.34)	<0.001	<0.001	0.031
FBG (mmol/L)	4.57 (3.89–5.55)	5.13 (3.57–6.47)	5.29 (4.26–6.44)	5.68 (4.73–6.61)	0.004	0.04	<0.001
FINS (mIU/L)	4.63 (3.76–5.88)	9.00 (7.33–10.43)	8.57 (7.72–9.53)	16.75 (15.31–18.17)	<0.001	<0.001	<0.001
HbAlc (%)	5.39 (4.89–6.11)	5.64 (4.85–6.32)	5.46 (4.67–6.35)	5.67 (5.06–6.26)	0.077	0.164	0.478
HOMA-*β*	81.50 (74.95–90.92)	118.26 (108.40–126.65)	94.28 (87.02–102.42)	156.6 (142.12–170.83)	<0.001	<0.001	<0.001
HOMA-IR	0.98 (0.92–1.05)	1.99 (1.83–2.13)	2.03 (1.90–2.17)	4.19 (3.95–4.43)	<0.001	<0.001	<0.001

*Note*. BMI, body mass index; WHR, waist-to-hip ratio; TG, triglyceride; TC, total cholesterol; LDL-C, low density lipoprotein cholesterin; HDL-C, high density lipoprotein cholesterin; FBG, fasting blood-glucose; FINS, fasting insulin; HbAlc, glycosylated hemoglobin; HOMA-*β*, homeostasis model assessment of beta cell function index; HOMA-IR, homeostasis model assessment of insulin resistance. *P*_a_, PCOS-nonobese group vs control-obese group; *P*_b_, PCOS-obese group vs control-obese group; *P*_c_, PCOS-obese group vs PCOS-nonobese group.

**Table 3:** Correlation analysis between serum miR-141-3p expression and clinical parameters in PCOS patients.

Parameters	PCOS-nonobese (*N* = 57)		PCOS-obese (*N* = 43)	
	Pearson *r*	*P* value	Pearson *r*	*P* value
Age (years)	/	0.974	/	0.557
BMI (kg/m^2)^	/	0.972	/	0.557
WHR	/	0.279	−0.427	0.004
TG (mmol/L)	−0.326	0.013	−0.338	0.026
TC (mmol/L)	−0.269	0.043	−0.432	0.004
LDL-C (mmol/L)	/	0.304	/	0.233
HDL-C (mmol/L)	/	0.091	0.434	0.004
FBG (mmol/L)	/	0.306	−0.421	0.005
FINS (mIU/L)	−0.47	<0.001	−0.656	<0.001
HbAlc (%)	−0.413	0.001	−0.55	<0.001
HOMA-*β*	−0.538	<0.001	−0.584	<0.001
HOMA-IR	−0.519	<0.001	−0.457	0.002

*Note*. BMI, body mass index; WHR, waist-to-hip ratio; TG, triglyceride; TC, total cholesterol; LDL-C, low density lipoprotein cholesterin; HDL-C, high density lipoprotein cholesterin; FBG, fasting blood glucose; FINS, fasting insulin; HbAlc, glycosylated hemoglobin; HOMA-*β*, homeostasis model assessment of beta cell function index; HOMA-IR, homeostasis model assessment of insulin resistance.

**Table 4:** Changes of clinical parameters in PCOS-obese patients during the 3-month intervention of fat reduction.

Parameters	PCOS-obese	Fat reduction intervention	*P* value
	(*N* = 43)	(*N* = 43)	
BMI (kg/m^2^)	28.55 (25.43–30.60)	27.39(24.63–30.54)	0.04
WHR	0.92 (0.82–1.02)	0.85 (0.77–0.94)	<0.001
TG (mmol/L)	1.51 (1.04–2.21)	1.42 (0.93–1.99)	0.076
TC (mmol/L)	1.30 (0.91–1.89)	1.22 (0.71–1.80)	0.104
LDL-C (mmol/L)	2.52 (1.99–2.91)	2.01 (1.66–2.41)	<0.001
HDL-C (mmol/L)	0.64 (0.17–1.34)	0.60 (0.07–1.20)	0.286
FBG (mmol/L)	5.68 (4.73–6.61)	5.43 (4.46–6.54)	0.265
FINS (mIU/L)	16.75 (15.31–18.17)	9.41 (8.09–10.92)	<0.001
HbAlc (%)	5.67 (5.06–6.26)	5.55 (5.04–6.13)	0.445
HOMA-*β*	156.6 (142.12–170.83)	94.78 (84.4–106.63)	<0.001
HOMA-IR	4.19 (3.95–4.43)	2.31 (2.10–2.54)	<0.001

*Note*. BMI, body mass index; WHR, waist-to-hip ratio; TG, triglyceride; TC, total cholesterol; LDL-C, low density lipoprotein cholesterin; HDL-C, high density lipoprotein cholesterin; FBG, fasting blood glucose; FINS, fasting insulin; HbAlc, glycosylated hemoglobin; HOMA-*β*, homeostasis model assessment of beta cell function index; HOMA-IR, homeostasis model assessment of insulin resistance.

## Data Availability

All the data generated or analyzed during this study are included in this published article.
